# What are emergency ambulance services doing to meet the needs of people who call frequently? A national survey of current practice in the United Kingdom

**DOI:** 10.1186/s12873-019-0297-3

**Published:** 2019-12-28

**Authors:** Helen A. Snooks, Ashrafunnesa Khanom, Robert Cole, Adrian Edwards, Bethan Mair Edwards, Bridie A. Evans, Theresa Foster, Rachael T. Fothergill, Carol P. Gripper, Chelsey Hampton, Ann John, Robin Petterson, Alison Porter, Andy Rosser, Jason Scott

**Affiliations:** 1 Swansea University Medical School, Singleton Park, Swansea, SA1 8PP UK; 2 West Midlands Ambulance Service, Trust Headquarters, Millennium Point, Waterfront Business Park, Waterfront Way, Brierley Hill, West Midlands DY5 1LX UK; 3 Division of Population Medicine School of Medicine, Cardiff University, Cardiff, CF10 3AT UK; 4 East of England Ambulance Service, Bury St Edmunds, Suffolk UK; 5 London Ambulance Service, 220 Waterloo Rd, Lambeth, London, SE1 8SD UK; 6 Welsh Ambulance Services NHS Trust Headquarters, Ty Elwy, Unit 7, Ffordd Richard Davies, St Asaph Business Park, St Asaph, Denbighshire LL17 0LJ UK; 7 Northumbria University, Sutherland Building, Newcastle-upon-Tyne, NE1 8ST England

**Keywords:** Emergency services, Calling frequently, Survey, Case management

## Abstract

**Background:**

Emergency ambulance services are integral to providing a service for those with unplanned urgent and life-threatening health conditions. However, high use of the service by a small minority of patients is a concern. Our objectives were to describe: service-wide and local policies or pathways for people classified as Frequent Caller; call volume; and results of any audit or evaluation.

**Method:**

We conducted a national survey of current practice in ambulance services in relation to the management of people who call the emergency ambulance service frequently using a structured questionnaire for completion by email and telephone interview. We analysed responses using a descriptive and thematic approach.

**Results:**

Twelve of 13 UK ambulance services responded. Most services used nationally agreed definitions for ‘Frequent Caller’, with 600–900 people meeting this classification each month. Service-wide policies were in place, with local variations. Models of care varied from within-service care where calls are flagged in the call centre; contact made with callers; and their General Practitioner (GP) with an aim of discouraging further calls, to case management through cross-service, multi-disciplinary team meetings aiming to resolve callers’ needs. Although data were available related to volume of calls and number of callers meeting the threshold for definition as Frequent Caller, no formal audits or evaluations were reported.

**Conclusions:**

Ambulance services are under pressure to meet challenging response times for high acuity patients. Tensions are apparent in the provision of care to patients who have complex needs and call frequently. Multi-disciplinary case management approaches may help to provide appropriate care, and reduce demand on emergency services. However, there is currently inadequate evidence to inform commissioning, policy or practice development.

## Background

Demand on emergency ambulance services is growing at an unsustainable rate [1]. The volume of emergency calls to ambulance dispatch centres in England doubled from 4.72 million in 2001/02, to 9 million in 2014/15, presenting major operational challenges in a time of constrained spending on health care [2, 3]. Media attention and public concern are high [4]. Recent United Kingdom (UK) policy calls for a whole systems approach to care, with new care pathways and greater clinical autonomy within the ambulance service to reduce pressures on Emergency Departments (EDs) [5, 6].

While emergency services remain integral to providing a service for those with unplanned urgent and life-threatening health conditions, high use of the service (includes calls to the ambulance service, EMS attendance or conveyance to ED) repeatedly by a minority of patients is a concern [7]. Their unresolved needs place pressure on the emergency ambulance service, which was designed to respond to patients with time-critical needs for clinical intervention, rather than to manage long-term care needs. Lack of pathways for referral and limited service availability, particularly out-of-hours, restrict the ability of ambulance personnel to direct patients to other services when appropriate. Patients may therefore attend ED, with lengthy waiting times and, potentially their needs can remain unresolved.

Definitions have varied and terminology is contested [7, 8] but UK ambulance services agreed in 2013 that people who make five or more calls per month or 12 calls over a three month period should be classified as ‘Frequent Callers’ [9]. In London alone, 1.7 million emergency calls were received in 2014–15. During this period 1622 people meeting the ‘Frequent Caller’ criteria generated 49,534 ambulance attendances, at a cost of £4.4 million to the London Ambulance Service (LAS) [1]. A similar story is repeated around the country [10, 11].

People who call the emergency ambulance service frequently may be experiencing falls, mental health problems, self-harming, misusing substances including alcohol, or living with chronic conditions. They are often vulnerable, lonely, living in poverty and experiencing a lower quality of life than the general population [7, 8, 12–15].. Patients who make high use of emergency healthcare – in particular the ED - experience higher mortality rates than the general population [16].

In England, national policy [17] recognises the challenges of responding to patients who access the ED repeatedly, and commissioners now require ambulance services to have management strategies in place for people who call frequently [18, 19]. International evidence indicates improved patient care and service delivery where case management is used in emergency care settings [20]. Some preliminary research on case management for this patient group in the UK reported significant reductions in calling following the introduction of case management in one area of London, although the sample size was small (*n* = 110) and the study design was an uncontrolled before and after comparison [7]. However, the extent and nature of services adopted across the UK’s ambulance service regions is currently unknown.

In this paper we present results of a national survey of current practice in relation to the management of people who call the ambulance service frequently. Our objectives were to describe: service-wide and local policies or pathways; numbers of people classified as Frequent Caller; and results of any audit or evaluation.

## Methods

### Design

Survey study using a structured questionnaire for completion by email and supplemented by telephone interviews. All participants firstly emailed their consent to participate in telephone interviews and also provided verbal consent prior to the interview taking place. This study did not require ethical approval from the NHS Health Research Authority (HRA) or Swansea University ethics committee. We used the HRA decision tool to determine need for ethics approval.

### Setting

UK ambulance services are funded centrally by the National Health Service consisting of services in England (*n* = 10), Wales (*n* = 1), Scotland (*n* = 1) and Northern Ireland (*n* = 1). Each ambulance service covers a wide geographical region, with separate commissioning arrangements within those service areas, totalling 218 groups responsible for commissioning services. Services commissioned within each local area include primary care, planned hospital care, rehabilitative care, urgent and emergency care (including out-of-hours), community health, mental health and learning disability services.

Ambulance services provide 999 emergency ambulances, rapid response vehicles, First Responders and patient transport services. Ambulance vehicles are required to carry a wide range of equipment including intravenous drips, drugs, oxygen and heart defibrillators. All services are expected to meet national performance targets to improve response times. Most air ambulance services in the UK are not part of the NHS and are funded through charitable donations.

Ambulance service staff are trained to a very high level, which enables them to deal with any aspect of emergency care, from minor injuries to cardiac arrest, or multiple casualties sustained in serious road traffic accidents. Ambulance crews typically include an emergency care assistant and a paramedic or an advanced paramedic practitioner.

### Data collection and participants

The questionnaire included open questions related to policies and pathways that were in place across the service and any local variations. We asked for details about who was involved in any cross-service partnerships or Multi-Disciplinary Team (MDT) meetings. We also asked about the volume of calls classified as Frequent Caller and whether the service had undertaken any audit or evaluation. We contacted the designated ‘Frequent Caller’ leads at all 13 UK ambulance services by email initially, with telephone follow up to complete the survey and clarify emailed responses (AK, CH).

### Analysis

We analysed responses (HS and AK) using a descriptive and inductive thematic approach [21] to data analysis where theoretical perspectives are informed by the interpretation of raw data. HS and AK read through the survey responses several times searching for reported commonalities and differences between services and systematically coded the data to draw out themes and categories to illustrate emerging concepts. Through discussion, these themes and categories were further refined and clustered into codes and sub-codes concerning policies and volume, care provision, perceived causes of high use and resources available.

### Research team

The team had multi-disciplinary and specialist input including paramedics, ambulance service managers, social care managers, public health and primary care clinicians, patients and methodologists. Patients worked alongside stakeholder and academic partners to oversee conduct of this work, consider implications of survey findings and how they could inform an application for further research [22, 23].

## Results

### Response rate

We received email and telephone responses from 12 of 13 UK ambulance services. Five ambulance services responded to our emailed survey questions, however we were able to collate a more detailed response from all 12 services through follow-up telephone calls. We received no response to our emails from one service.

We report on four themes identified from our analysis of the survey data. These include policies and volume; care provision; perceived causes of high use and resources available.

### Policies and volume

Most respondents (*n* = 10) reported that a service-wide policy was in place, with all services using the nationally agreed criteria for ‘Frequent Caller’ classification and compiling a list of patients meeting this definition. These callers are flagged on the electronic ambulance dispatch system so that subsequent calls are identified. Some services provided data about the scale of the problem – ranging from 600 to 900 patients included on lists each month. Although each service held data on patients, there was little reported in the way of formal audit or evaluation. Two services reported that they were taking no specific actions in relation to this patient group. Responses are summarised in Table 1.

**Table 1:** A summary of responses to a UK wide survey of ambulance service current policies and practice in relation to the management of the care of people who make frequent emergency calls

*Service*	*Service-wide policy*	*Local policies/pathways*	*Call volume*	*Audit/ Evaluation*
*1*	*Service wide policy in place* *National definitions for ‘Frequent Caller’ used* *Aim – to discourage people from calling*	*One area of service has an initiative which started in 2015* *Aim – to identify triggers which lead to the patient making the call* e.g. *adverse childhood events, mental health, drug and alcohol dependency*	*Not reported*	*No audit or evaluation reported; Frequent Caller lead reviews cases on four monthly basis*
*2*	*Service wide policy updated 2017* *National Definitions for ‘Frequent Caller’ used* *Aim – to reduce further calls to emergency ambulance service*	*One area of ambulance service has initiative started in 2017.* *Aim – to work together to ascertain any unmet needs with a view to reducing demand*	*346 patients on active management plans at time of response*	*Auditing at individual case level, in process of developing evaluation process on larger scale*
*3*	*Service wide policy in place* *National definitions for Frequent Caller used* *Aim – to close cases (take the person off the frequent caller list) and reduce calls*	*Several areas participate in MDT meetings.* *No specific aim given*	*600 patients a month, team involved with 50 callers per month*	*None reported*
*4*	*Service wide policy in place* *National definitions for ‘Frequent Caller’ used* *Aim- to reduce or prevent people from calling*	*In several areas cross service initiatives are in place.* *Aim – to work as a multi-agency team to reduce demand and provide the patient with the most appropriate care to meet their complex needs*	*900 people per month are classified as ‘Frequent Caller’*	*No audit or evaluation undertaken*
*5*	*Service wide developed policy developed in 2017* *National definitions for Frequent Caller used* *No specific aim reported*	*In one area cross-service initiative started in 2017* *No specific aim reported*	*200 calls per month*	*No audit or evaluation reported*
*6*	*Service wide policy in place since 2013, currently being updated* *National definitions for ‘Frequent Caller’ used No specific aim reported*	*Additional programme in one area targeted at patients with mental health problems – started in 2016* *No specific aim reported*	*No data provided*	*No audit or evaluation reported*
*7*	*Service wide policy in place* *National definitions for ‘Frequent Caller’ used* *Aim – to reduce calls and address reasons for calling Actions for callers meeting defined threshold*	*No variations reported by area*	*2500 people classified as Frequent Caller per year*	*No audit or evaluation reported*
*8*	*Service wide policy since 2007* *National definitions for ‘Frequent Caller’ used* *No explicit aim given*	*Several areas have cross service initiatives in place* *Aim – to ensure that patients have access to the most appropriate care pathway. Usually callers have an exacerbation of an underlying chronic illness over a short period of time and do not know which service to access; or they have recently left hospital (geriatric) or prison; have a mental health condition; have recently experienced a bereavement or have a drug or alcohol problem*	*No data given*	*No audit or evaluation reported*
*9*	*Service wide policy since 2013* *National definitions for ‘Frequent Caller’ used* *No specific aim given*	*In one area a service has been set up which uses the GP neighbourhood model* *Aim – to identify why patients are calling and see what support can be put in place to support them*	*No figures given*	*No audit or evaluation reported*
*10*	*Service wide policy since 2009* *National definitions for ‘Frequent Caller’ used* *Aim – to reduce calls and ED attendance*	*One area has a new pilot service which started in 2017* *Aim – to reduce calls and reduce ED attendance*	*700 calls per month*	*Pilot schemes audited on monthly basis*
*11*	*No response*			
*12, 13*	*Services responded that the survey was not applicable, little was done and service 12 stated that no letters were sent to patients*			

### Care provision

In many areas, service-wide policy was to contact the person who has reached the threshold of the Frequent Caller classification, usually by letter. In the letter the patient is encouraged to contact their General Practitioner (GP) to try to address their healthcare problems. The GP is also alerted to the classification of one of their patients as a Frequent Caller. A care plan may be written within the ambulance service, for use in the ambulance dispatch centre. In this case, any further calls are triaged and may be passed to a clinician in the dispatch centre for advice over the telephone, with referral to other services if appropriate and available. Anti–social behaviour arrangements may be put in place (services 4, 8). Two services reported that home visits may be made by an ambulance service operational or clinical manager with an appropriate health care professional e.g. mental health, drugs and alcohol service or the police (services 4, 8). Service 4 described the drafting of a 12 month ‘Acceptable Behaviour Contract’ by the multi-disciplinary team, for sharing with the caller during a home visit. “Inappropriate use of services” would be discussed during that visit. In this service “if all other measures failed” a “restricted send” policy could be put into place – to only send an ambulance in the case of a life-threatening condition. An escalation option was also reported by service 4, of referral to the police for action to be taken under the Misuse of Communications Act 2003 [24]. Monitoring of safeguarding issues was reported by one service (9). The explicit aim of these ‘within-service’ models was described as discouraging further calling, and to manage further calls without the dispatch of an emergency ambulance.

Despite reporting service-wide policy, nine services reported also having local cross-service multi-disciplinary initiatives in some areas (example presented in Box 1). Nine services reported that they had implemented cross-sector multi-disciplinary case management models in at least one area (services 1,2,3,4,5,6,8,9,10), with one reporting that case management was in place across the entire service area (service 7). Case management models varied locally, in some cases being available only to a subgroup of the population e.g. ‘persistent’ or ‘non-engaged’ callers (services 3,4,10) or callers with mental health problems (services 6,10). Multi-disciplinary case management meetings had varied attendance, but typically included clinical and operational representatives from: Ambulance service, Emergency Department, primary care, Community Mental Health Trust, Community matron/District nurse service, Clinical Commissioning Group, Social services, housing department, Fire Service, Police, Patient Advocacy Services/voluntary sector, and Occupational Therapy services. Patients may be invited to attend these meetings to discuss their issues with care providers across some services (services 1, 4), whilst other services may hold meetings to discuss patient needs in their absence (service 2). Some multi-disciplinary meetings were chaired and hosted by the ambulance services (service 3); and in other cases the ambulance service representative attended the meeting, which may include planning the care of patients who make high use of other services such as the emergency department (services 2,5). Within the case management approach two services (services 8, 10) reported visiting patients at home whenever possible to better understand their needs. Service 10 reported carrying out home visits to undertake a holistic assessment of needs; with provision of equipment, support to attend appointments, medication reviews and referral to other agencies as available options to try to meet patients’ needs and reduce further emergency responses from the ambulance service.

**Box 1:** Example of variation between areas within one service

Service-wide policy	Local cross-service multi-disciplinary initiative
Aim – to discourage people classified as Frequent Caller from further calling Actions for callers meeting defined threshold: • Clinical Support Officer (CSO) contacts patient by letter stating they have called the emergency ambulance service more than normal and should seek help from their GP, contact number provided within letter for patient to talk to ambulance service manager; and calls or sends letter to GP to make them aware patient is calling the emergency ambulance service frequently • If calls persist, CSO may contact other services to intervene and support patient • CSO writes individual care plan which is shared with the call centre clinical team. When the patient calls (s) he is triaged to a clinician in the call centre rather than sending an ambulance • If caller persists then (s) he may be referred to the police and court	Aim – to identify triggers which lead to the patient making the call e.g. adverse childhood events, mental health, drug and alcohol dependency Actions for callers meeting defined threshold: • CSO speaks to GP or practice manager first • Patient is discussed at monthly MDT meeting. Around 50 patients may be discussed at one meeting, 10–15 of these making high use of ambulance service. MDT is used to provide a network of support for the patient and to address their needs through multi-agency working. Professionals see it as part of their role to support people who frequently access the ambulance service, police or ED. Agencies involved: police, ED, Out of Hours primary care, voluntary sector, social services • Patient allocated to appropriate agency to lead on care planning and provision. A care plan is created and shared so that any agency contacted by the patient knows what has been agreed • Patient interviewed individually to assess need • If calls persist or patient has an anti-social behaviour order, the CSO visits the patient along with a police officer or representative from the ED

### Perceived causes of high use

Services reported that sometimes people make high use of the emergency ambulance service in a period of change or crisis e.g. exacerbation of underlying chronic illness, following discharge from hospital, recent bereavement or release from prison. Service 8 reported that most calls arose when patients did not know how to access appropriate services, and that in some cases contact with the patient, identification of underlying issues and discussions or referral may resolve the immediate problem of frequently calling the emergency ambulance service.

### Resources available

In some cases one or more Clinical Support Officers or the Frequent Caller lead (usually paramedic) were allocated to work on resolving these cases (services 1,2,6,9); in other services this work was undertaken by local operations managers (services 3,4,5) or a mixed team (services 7,10). In one service National Health Service (NHS) 111, a telephone based 24 h health information and advice line, managed this caseload (9). In three services resources for tackling this workload were not clearly reported (services 8,12,13).

## Discussion

### Summary of findings

The management of people who make frequent calls to ambulance services is a priority for UK ambulance services, supported by national and local policy. All responding ambulance services had a designated ‘Frequent Caller’ lead and kept data about calls made by people classified as Frequent Callers, using nationally agreed definitions. Models of care for these patients varied widely between and within services, from ‘within-service’ flagging and efforts to discourage further calls to partnership working across services through multi-disciplinary team meetings in a case management approach. Although data were available related to volume of calls and number of callers meeting the threshold for definition as Frequent Caller, no formal audits or evaluations were reported.

### Limitations

We achieved a high response rate, although very little detail was provided by two devolved nations within the UK, and one regional service did not respond at all. We are unaware of any differences between responding and non-responding services in terms of the management of the care of people who call frequently. We used a semi-structured survey approach with responses by email or telephone. Detail varied by respondent and there were inconsistencies in responses provided.

### Implications

Tension is apparent between management of this caseload to reduce pressure on the ambulance service and management of these callers in response to their needs. In some cases the processes for managing the care of people who call frequently were led by staff in operational management roles, in others care was clearly within the remit of clinical members of staff. The aims of the services reflected this tension. Where the response was primarily ‘within-service’, aims tended to focus on reducing calls, with an emphasis on managing the behaviour of people making high use of the emergency ambulance service. Referral of callers to the police for anti-social behaviour management or prosecution was reported as an option. By contrast, the cross-service partnerships used a ‘case management’ type model, seeking to resolve callers’ clinical, social or emotional needs. Reflecting this tension, multi-disciplinary teams included clinicians, health service managers, voluntary sector representatives and the police.

Previous research has shown that people who frequently call emergency ambulance services may be vulnerable and unable to access more appropriate services, such as primary care [5, 8, 11–14]. They appear to seek help through this route because other available services do not meet their complex needs, particularly during evenings and weekends. However, the emergency ambulance route to help-seeking may often be unsuccessful and inefficient, for them individually and for the NHS. It has potential consequences also for other patients who do not get the speedy response required when in urgent need, as resources are tied up with patients who may not need acute care. There is a conflict between the time required for appropriate assessment, onward referral, management planning and operational pressures of emergency work. Services are being pressured by commissioners to reduce times spent on scene and to reduce conveyance to ED, again reflecting the tensions inherent in provision of care to patients with complex needs. Over the longer term these policies and pulls should work together if effective, but in the short term there may be increased on-scene times which can be regarded as an additional burden on the emergency care service. Multi-disciplinary case management approaches with strong emergency ambulance service involvement may reduce demand on emergency services and emergency hospital admissions because people are effectively treated [25–27].

### Future research

The introduction of case management has potential to achieve safe and equitable out-of-hospital care for patients who call the emergency ambulance service frequently, whilst avoiding shifting patients to another part of the emergency care system without their needs being met. However, additional research is required to determine whether case management is effective in practice in this setting [20, 28]. The recently funded quasi-experimental STRETCHED study (National Institute of Health Research (NIHR) HS&DR 180302) investigates the effectiveness of case management approaches for people who make high use of the emergency ambulance service. We will investigate what helps case management to work, and what hinders the implementation and effective functioning of this model.

Results of the STRETCHED evaluation will inform policy and practice to support people who make high use of the emergency ambulance service. Many services have implemented case management approaches in at least one area, but with limited evidence of effectiveness of efficiency.

## Conclusions

Ambulance services are under pressure to meet challenging response times for high acuity patients. Tensions are apparent in the provision of care to patients who have complex needs and call frequently. Within service care focused mainly on reducing calls whilst multi-disciplinary case management aimed to resolve clinical and emotional needs of patients. However, there is currently inadequate evidence to inform commissioning, policy or practice development.

## Data Availability

The data that support the findings of this study are available from the corresponding author upon reasonable request.

## Abbreviations

AK: Ashrafunnesa Khanom
CH: Chelsey Hampton
CSO: Clinical Support Officer
ED: Emergency Department
GP: General Practice
HS: Helen Snooks
MDT: Multi-Disciplinary Team
NHS: National Health Service
NIHR: National Institute of Health Research
UK: United Kingdom
